# *Pycnoscelus surinamensis* cockroach gut microbiota respond consistently to a fungal diet without mirroring those of fungus-farming termites

**DOI:** 10.1371/journal.pone.0185745

**Published:** 2017-10-03

**Authors:** Callum Richards, Saria Otani, Aram Mikaelyan, Michael Poulsen

**Affiliations:** 1 Centre for Social Evolution, Section for Ecology and Evolution, Department of Biology, University of Copenhagen, Copenhagen East, Denmark; 2 Department of Biological Sciences, Vanderbilt University, VU Station B, Nashville, TN, United States of America; 3 Department of Pathology, Microbiology, & Immunology, Vanderbilt University, VU Station B, Nashville, TN, United States of America; Cairo University, EGYPT

## Abstract

The gut microbiotas of cockroaches and termites play important roles in the symbiotic digestion of dietary components, such as lignocellulose. Diet has been proposed as a primary determinant of community structure within the gut, acting as a selection force to shape the diversity observed within this “bioreactor”, and as a key factor for the divergence of the termite gut microbiota from the omnivorous cockroach ancestor. The gut microbiota in most termites supports primarily the breakdown of lignocellulose, but the fungus-farming sub-family of higher termites has become similar in gut microbiota to the ancestral omnivorous cockroaches. To assess the importance of a fungus diet as a driver of community structure, we compare community compositions in the guts of experimentally manipulated *Pycnoscelus surinamensis* cockroaches fed on fungus cultivated by fungus-farming termites. MiSeq amplicon analysis of gut microbiotas from 49 gut samples showed a step-wise gradient pattern in community similarity that correlated with an increase in the proportion of fungal material provided to the cockroaches. Comparison of the taxonomic composition of manipulated communities to that of gut communities of a fungus-feeding termite species showed that although some bacteria OTUs shared by *P*. *surinamensis* and the farming termites increased in the guts of cockroaches on a fungal diet, cockroach communities remained distinct from those of termites. These results demonstrate that a fungal diet can play a role in structuring gut community composition, but at the same time exemplifies how original community compositions constrain the magnitude of such change.

## Introduction

Gut microbes have had a significant impact on animal evolution and play a diverse range of functional roles within their symbiotic hosts [1, 2]. Complex gut microbiotas are found in species ranging from mammals to insects and have crucial roles in digestion, immunity, and development [3, 4]. Understanding the mechanisms that govern the ecology and evolution of complex microbial communities is important to gain further insight into the development of these mutualistic (beneficial) symbioses [5–7]. Research into the microbiology of insect symbionts has increased over recent years with advances in sequencing technologies that have helped identify the microbes dominating insect guts in, among others, *Drosophila*, honey bees and attine ants [3, 8–11]. Termite guts are of particular interest as they harbour diverse and unique microbial populations, particularly in the hindgut that is characterized by the breakdown of lignocellulose, and acts as a major “bioreactor” characterized by the low redox potential and the accumulation of hydrogen [12–15].

Termites are eusocial cockroaches that evolved from an omnivorous cockroach ancestor more than 150 million years ago, accompanied by the specialisation to a wood-feeding lifestyle [16, 17]. The transition from an omnivorous to a wood-feeding life style was enabled by the acquisition of cellulolytic flagellates that can still be observed as predominant members of the enlarged hindguts in primitive “lower” termites and their cockroach sister group, the Cryptocercidae [18–20]. The subsequent loss of gut flagellates in the Termitidae led to the radiation of the so-called “higher” termites and dietary diversification as this group evolved to feed on a variety of lignocellulosic food sources with the aid of a completely prokaryotic gut microbiota [20,21].

Diet has been suggested as a major driver of bacterial community structure in the guts of higher termites, with major dietary shifts and diversification being associated with compositional changes of the gut microbiota [17, 22–23]. Convergence of bacterial community structure would therefore be expected to occur between species that share a dietary specialization, particularly in species with a highly specific diet, such as in the fungus-cultivating Macrotermitinae, where the fungal genus *Termitomyces* is the main food source [24]. This symbiosis has allowed the termite subfamily to become of major importance in plant degradation and nutrient cycling within its ecological range, with members of the Macrotermitinae estimated to consume more than 90% of dry wood litter in African savannahs [25].

A shift to a proteinaceous fungal diet of the fungus-farming termites may be responsible for a convergence of community structure between this specialized group and their omnivorous non-eusocial cockroach relatives [21, 22]. Otani et al. [22] sampled guts from nine species of fungus-farming termites and found that the Macrotermitinae associate with a core gut microbiota that is more similar to each other and to cockroach gut communities than to other termites. They observed a resurgence of bacterial taxa that prevail in cockroaches, with a shared predominance of Bacteroidetes and Firmicutes [21, 22], which are common in omnivorous animals and may have been promoted by the protein-rich fungal components of the fungus-farming termite diet [21, 26]. This suggests that the obligate association with *Termitomyces* has shaped the gut microbiota to be compositionally different to those of other termites [21, 22].

The dense microbial colonisation of the homologous hindgut in the cockroach *Shelfordella lateralis* and its microbial metabolite profile suggest that the hindgut is also the major site for microbial activity in cockroaches [26]. Cockroaches are amenable to dietary manipulation, and previous studies have shown the ability of diet to modulate gut community composition [27, 28]. Such approaches are limited in termites, because of their tighter dependence on gut microbes and because they in many cases are harder to manipulate in a laboratory setting. Here we test if a fungal diet can act as a selective force to alter the composition of microbiota in the gut of the litter-feeding cockroach *Pycnoscelus surinamensis*. By providing fungal material from a pure culture of *Termitomyces* sp. isolated from a fungus-farming termite nest, we mirror fungus feeding and use MiSeq sequencing of the 16S rRNA gene to compare bacterial community structure between cockroaches fed on increasing dietary proportions of dried *Termitomyces* biomass relative to a normal leaf-litter diet. We hypothesised that the cockroach gut microbiota composition would respond to an increasing proportion of fungal biomass in the diet, in such a way that it would more closely reflect the composition of fungus-growing termites.

## Materials and methods

### Study species

Individuals of the litter-feeding cockroach *Pycnoscelus surinamensis* were obtained from a commercial breeder [29]. *P*. *surinamensis* is a species of burrowing cockroach endemic to the Indomalayan region and is a common plant pest that has colonized New World tropical and sub-tropical regions due to its ability to reproduce quickly via thelytokous parthenogenesis; a process that produces functional female offspring from unfertilized eggs [30–32]. It is a member of the Blaberidae, a sister family to the combined termite, *Cryptocercus*, and Blattidae clade [33], placing it well to act as a model for termite evolution. The cockroaches were maintained at the University of Copenhagen in climate rooms at 27°C and 50% relative humidity. An initial stock population of ca. 1000 individuals was established and maintained throughout the experimental period in a plastic container (56x39x28cm) containing a soil and leaf litter substrate. The cockroaches were fed leaf litter, fruit, and vegetables three times per week and the substrate within the container was replenished weekly until three days before initiation of the feeding experiment.

### Diet experiment

**Fig 1:**
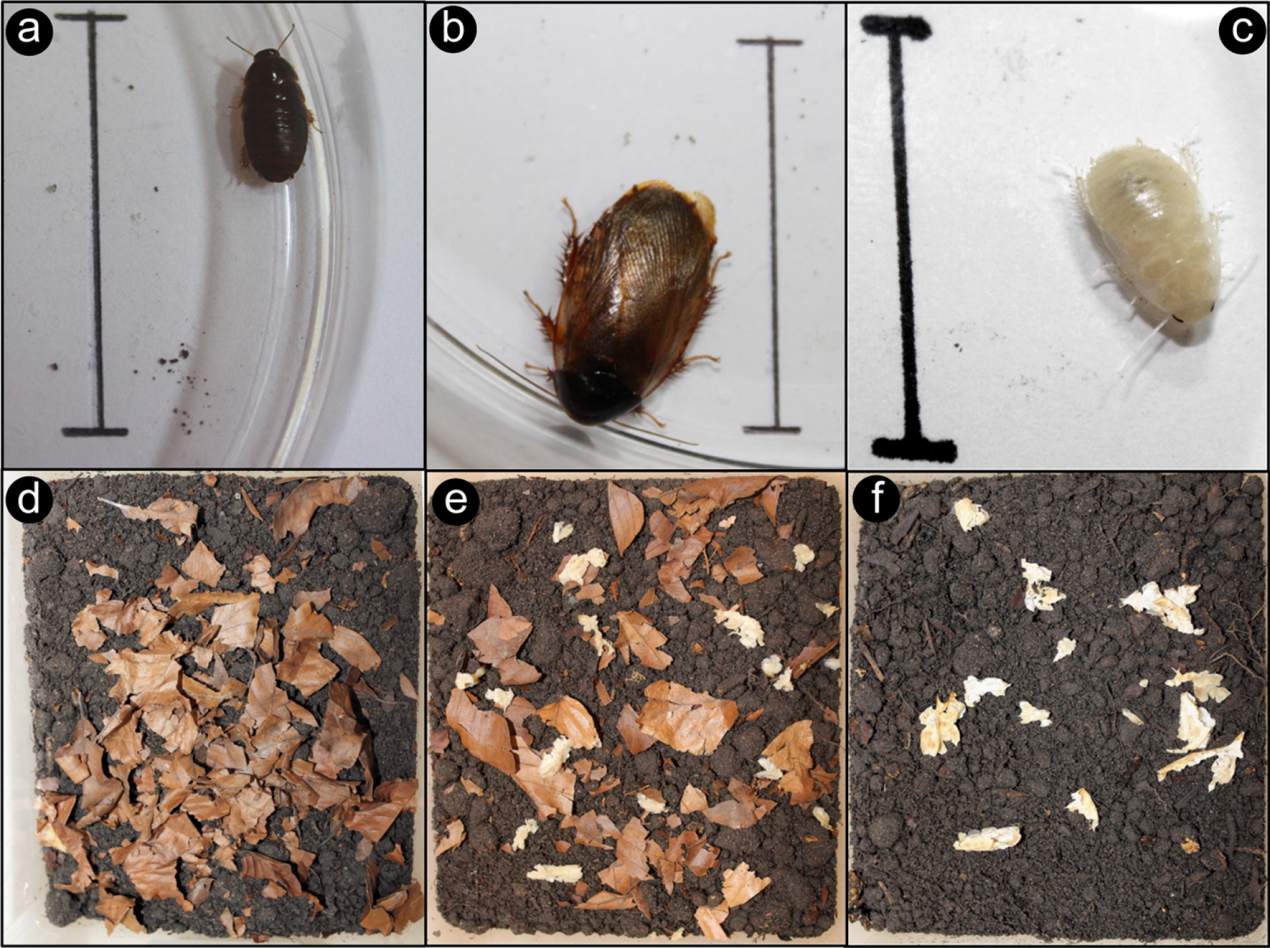
(a-c) A juvenile (a), an adult (b) and a newly moulted (c) *Pycnoscelus surinamensis* individual, the scale bar (3cm) was used to assess the size of cockroaches as a proxy for their age and therefore likelihood of moulting during the experiment. Individuals selected for the experiment were approximately 8–10mm in length. (d-f) Treatment boxes with diets consisting of 0% (d), 60% (e), and 100% (f) *Termitomyces* fungus, respectively.

After a two-week pre-feeding period, individuals within the holding container were exposed to a control diet of only leaf litter for 72 hours and juveniles were subsequently isolated into subsets of 50 cockroaches within smaller experimental containers (21x17x15cm). Juveniles were chosen to ensure the occurrence of at least two moults and subsequent restoration of the gut microbiota during the experimental period [34]. This process was expected to allow for the microbiota to change as a consequence of an altered diet and results in the cockroach appearing white for a short period, as its exoskeleton loses pigmentation after moulting (Fig 1C) [35]. This was observed and recorded during the experiment to enable the monitoring of the moulting process. Over a one-month treatment period, sub-populations were exposed to one of six diet regimes consisting of 0 to 100% dried fungal biomass obtained from a *Termitomyces* sp. isolated from the colony *Odontotermes* sp. Od127 [36]. *Termitomyces* was cultured on Potato Dextrose Agar (PDA, 39g/L PDA, 10g/L agar) and incubated at 27°C for at least 96 hrs to allow sufficient fungal growth, after which fungal material was harvested by scraping off mycelium, taking special care to avoid the medium. Harvested mycelium was dried at 56°C for four hours before being combined with the appropriate dry weight of leaf litter to produce the feed allowance for the treatment sub-populations. Sub-populations were provided with 1.5g of forage material, consisting of one of the following combinations (percentage-by-weight ratios) of dried leaf-litter to fungus material: 100:0, 80:20, 60:40, 40:60, 20:80 or 0:100 (Fig 1D). Each of the six dietary combinations was set-up in triplicate, yielding a total of 18 sub-populations, which were fed twice a week for a one-month treatment period. Uneaten food was removed before new provisioning to keep the leaf litter to fungus ratios as consistent as possible.

### Survival and behaviour surveys

Each subpopulation was surveyed twice a week and their foraging behaviour recorded to establish if the cockroaches consumed the fungal biomass. The number of juveniles, sub-adults and adults were counted for each sub-population at the end of the experiment to compare the well-being of cockroaches on different feeding regimes. Cockroach age was approximated based on size (Fig 1A–1C).

### Dissections and DNA extraction

For each sub-population, nine cockroaches were randomly picked for dissections, and these nine were randomly assigned to one of three technical replicates per sub-population. Because of the labour-intensiveness of the dissections, only 1/6^th^ of all cockroaches included in the experiment could be dissected in one day. Therefore, three sub-populations were randomly picked daily for dissections, while sub-populations yet to be dissected were maintained on their diet regime in order to avoid starvation and to presumably sustain a stable gut microbiota. Before dissection, cockroaches were subdued on ice for 20 minutes, after which they were placed dorsally on a sterile Petri dish, the head was removed, and the tergal area opened by coaxial removal of the legs, exposing the body cavity and allowing removal of the gut from the anus to the metathorax. The hindgut was separated from the whole gut while saturated in RNAlater® (Ambion®Thermo Fisher Scientific, Nærum, Denmark). Dissections were carried out under stereomicroscope (Wild M3C, Leica Microsystems, Ballerup, Denmark) using fine forceps and guts were stored at -20°C until DNA extraction using the DNeasy blood and Tissue kit (Qiagen, Germany), following the manufacturer’s instructions.

### Bacterial 16S rRNA PCR amplification and MiSeq sequencing

The V4 region of the 16S rRNA gene was amplified using the primers v4.SA504 and v4.SB711 [36]. The V4 region amplification was carried out using a dual indexing sequencing strategy [37], and the PCR mixture was prepared in 20 μl volumes, containing 11.85 μl sterile distilled water, 2μl of each primer (4.0 μM), 2 μl of 10x AccuPrime PCR buffer II (Life Technologies, Carlsbad, CA, USA), 2μl DNA template, and 0.15 μl AccuPrime High Fidelity Taq DNA polymerase (Life Technologies, Carlsbad, CA, USA). PCR conditions were 95°C for 2 minutes followed by 30 cycles of 95°C for 20 s, 55°C for 15 s, and 72°C for 5 min followed by 72°C for 10 min. Troubleshooting PCR was carried out with 2 μl of 1:10 diluted DNA template. Library normalisation was carried out using Life Technologies SequencePrep Normalization plate kit (Life Technologies, Carlsbad, CA, USA) following the manufacturer’s instructions. Sample concentration was measured using Kapa Biosystems Library Quantification Kit for Illumina Platforms (Kapa Biosystems, Wilmington, MA, USA) and the size of library amplicons was determined using Agilent Bioanalyser High Sensitivity DNA analysis kit (Invitrogen, Carlsbad, CA, USA). After selection of the most promising samples (S1 Table), they were subjected to sequencing on the Illumina MiSeq platform using MiSeq Reagent Kit V2 500 cycles [37].

### Sequence filtering and taxon classification

Raw flow grams from sequencing were analysed using Mothur v. 1.37.6 [38] and the standard operating procedure was followed as described at http://www.mothur.org/wiki/MiSeq_SOP[38]. Paired-end reads were assembled into contigs and subjected to several filtering steps in order to reduce PCR and sequencing errors. High-quality sequences were aligned against the manually curated reference database DictDb v. 3.0 [39]. This database was generated from the SILVA 102 non-redundant database with additional termite and cockroach gut 16S rRNA gene sequences added to improve classification resolution; it is available upon request [36]. Operational taxonomic units (OTUs) were calculated at the 2% species level classification and rarefaction curves based on a 97% sequence similarity cut-off were generated using the ‘Vegan’ statistical package for community ecology [40] in R version 3.3.3 [41].

### Analysis of gut community diversity and similarity between different fungal diets

Relative taxa abundances were calculated as the number of sequence reads per taxon for the 54 gut samples, after which the abundances for biological replicates were obtained from averaging the three technical replicates. Principal coordinates analysis (PCoA) to determine community similarity between three biological replicates per diet regime was performed in R [41], based on Bray-Curtis distances. PCoA loading values were used to assess the contribution of genus level-taxa to the patterns observed in a full comparison of all diet regimes, as described in [22].

The distribution of the most abundant taxa was further compared to data on gut community compositions in five colonies of the fungus-growing termite *Odontotermes* sp. obtained from [36]. Gut community alignments from the fungus-fed treatment samples were combined to alignments from *Odontotermes* sp. and assigned to taxa using the naïve Bayesian classifier ran against the manually curated reference database DictDb v. 3.0 [39]. We then visualised relative taxon abundance differences across the combined datasets in two PCoA analyses, including determining loading values to assess the contribution of genus-level taxa [22, 41]. The first PCoA included all OTUs identified in the cockroach treatment groups fed 0% and 100% fungal diets and gut communities in *Odontotermes* sp., and the second PCoA included only OTUs that were found in communities in termite and cockroach treatment groups fed 0% and 100% fungus. The latter was performed to explore whether the dissimilarity observed between cockroach and *Odontotermes* sp. guts (see below) was mainly due to the lack of overlapping bacterial taxa between the two.

## Results and discussion

### Mortality and behaviour surveys

**Fig 2:**
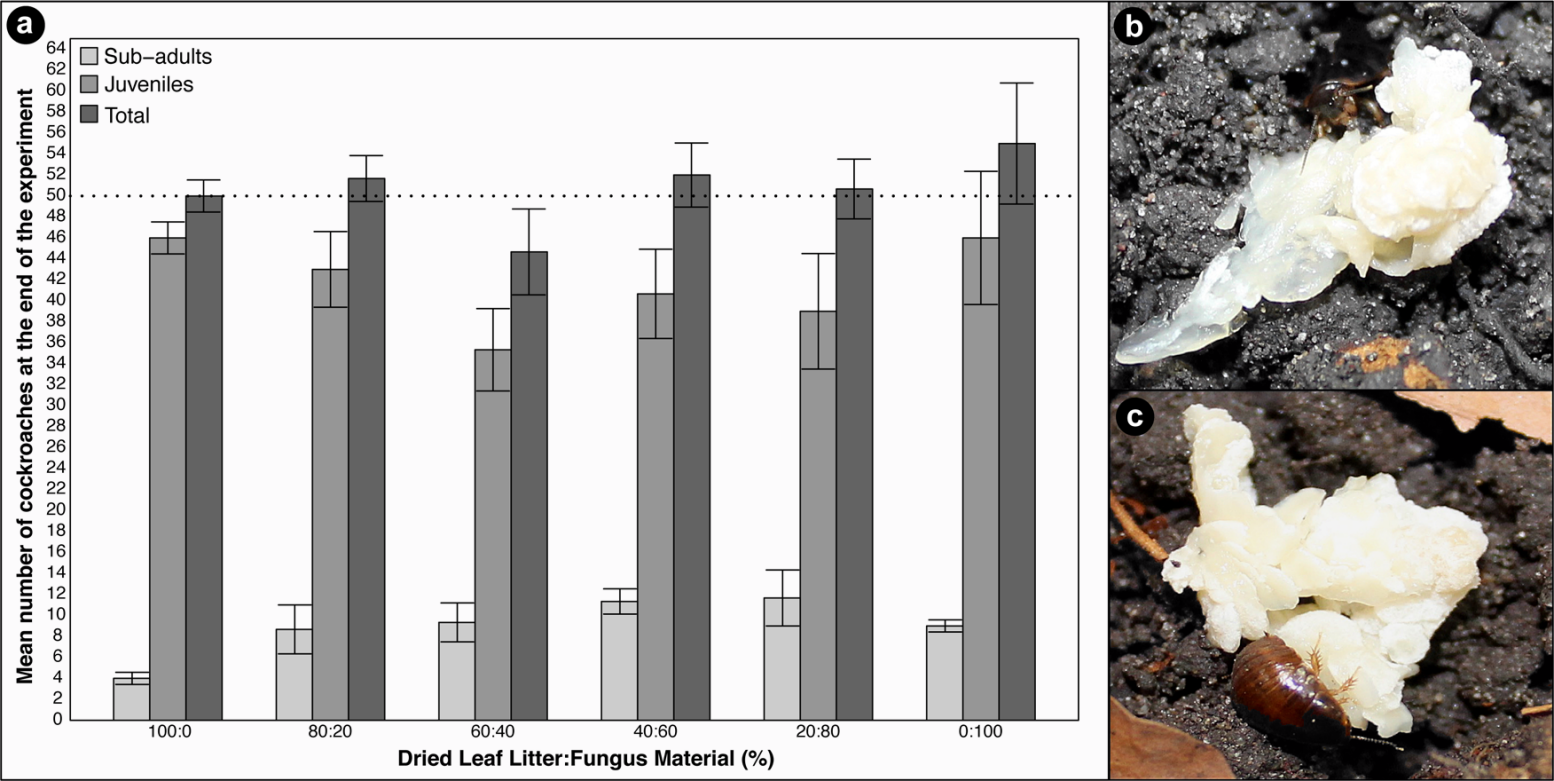
(a) Mean ± SE (n = 3) number of juvenile (grey), sub-adult (light grey) and total number of cockroaches (dark grey) within each diet regime remaining at the end of the experiment. No fully-grown adults were observed within the sub-colonies at the end of the experimental period. A population size of approximately 50 individuals (intersecting dotted line) was maintained in the majority of diet regimes, and no significant differences in survival were observed between different fungal ratios. (b) *P*. *surinamensis* sub-adult feeding on *Termitomyces* and (c) juvenile handling fungal material.

Behavioural observations indicated that *P*. *surinamensis* cockroaches were able to consume the provided fungal material with active feeding frequently observed throughout the duration of the experiment. The cockroaches would drag fungal material down into the soil after a short initial feeding period and occasionally feed on material on the soil surface (Fig 2B). Minimal fungal material was left untouched after feeding periods during the experimental period and individuals remained active in all diet regimes below the 20:80% regime. Moulting was frequently observed over the course of the experiment, with depigmented individuals being present in all diet regimes. Activity levels did appear to decrease in 20:80% and 0:100% fungus diets, where individuals moved at slower speeds and were at times found dormant within the soil substrate. However, this did not increase mortality, as the end numbers of cockroaches across all sub-populations were not significantly affected by diet (Cox Proportional-Hazards Regression; Wald χ^2^ = 1.15; df = 5; p = 0.9493) (Fig 2A). This suggests that there were no short-term negative effects due to fungus feeding, but more extensive longer-term experiments would be needed to explore if there are longer-time physiological or fitness effects.

### Illumina MiSeq data

**Fig 3:**
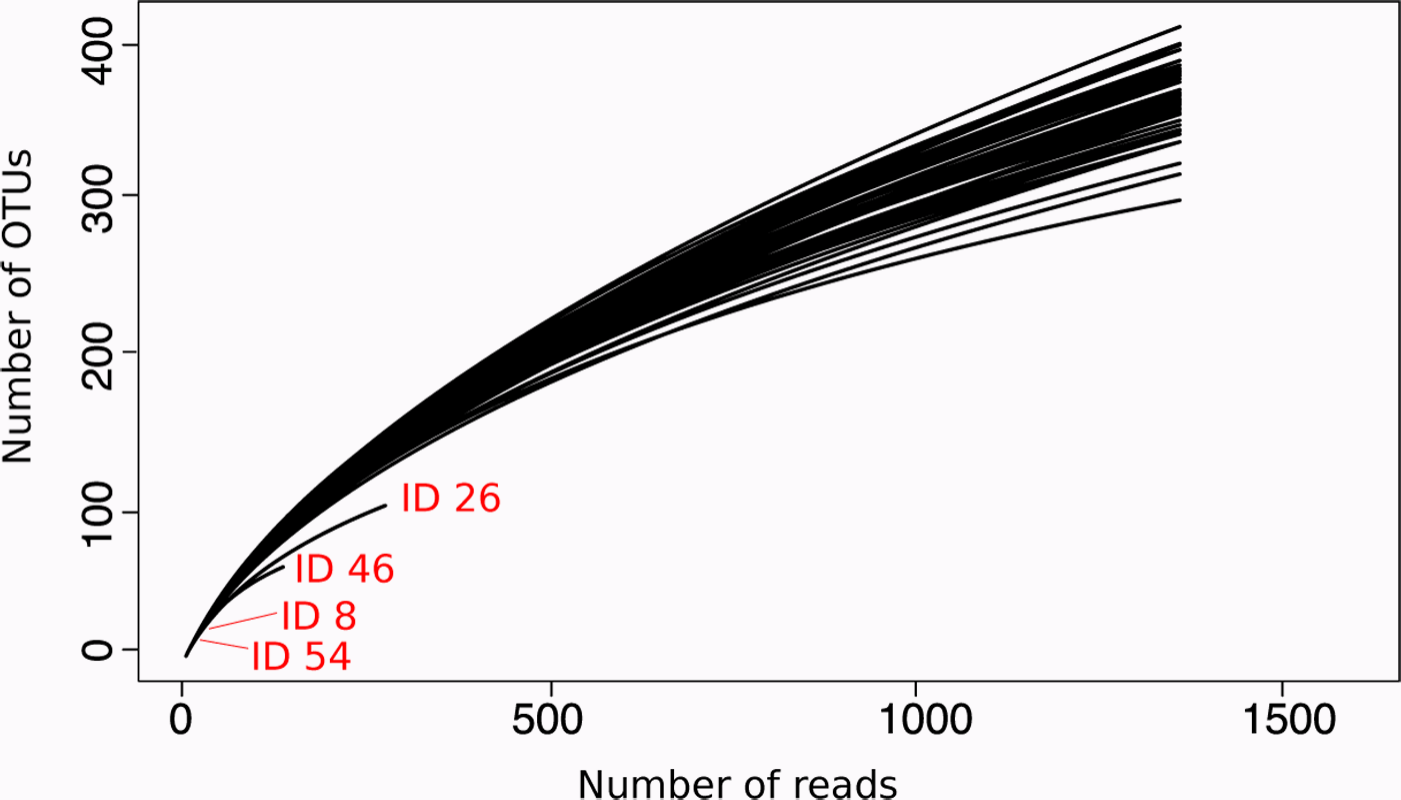
Rarefaction curves of sequence depth for the 54 gut samples [41]. Each curve represents the number of identified OTUs as a function of the number of sequenced reads after filtering. The samples ID 8 (0% fungus, replicate 3, technical replicate 2), ID 26 (40% fungus, replicate 3, technical replicate 2), ID46 (100% fungus, replicate 1, technical replicate 1) and ID 54 (100% fungus, replicate 3, technical replicate 3) were omitted from downstream analysis due to low sequence read count and subsequent poor coverage of bacterial communities. The remaining 50 samples had sufficient coverage and were used for analysis of community diversity and taxa abundances.

Rarefaction analysis showed sufficient coverage of all but four bacterial communities (IDs 8, 26, 46, and 54; S1 Table; Fig 3), so these were omitted from subsequent analyses with no loss of any gut microbiota sample. 16S rRNA gene sequencing of the remaining 50 cockroach gut samples generated from 9,541 to 12,267 high quality reads (mean±SE: 11,131±547) per sample (Table 1). A total of 3,145 unique OTUs at the 2% cut-off level were identified after filtering and sequence analysis (S2 Table). The number of genus-level taxa per sample ranged from 178 to 194 (average 184±2.25) (Table 1), with cockroaches fed on a 20:80% leaf litter:fungus regime harbouring the least. Shannon and Simpson diversity indices were however similar across all treatments (Table 1).

**Table 1:** The number of sequences after filtering of raw reads, the number of identified taxa, the percentage of reads successfully assigned to the phylum, family and genus levels (based on relative abundances) as well as the estimated richness and diversity indices for the bacterial communities (at 2% dissimilarity threshold).

			**Classification Success (%)**			**Diversity Indices**		
**Leaf litter: fungus**	**Number of sequences**	**Mean±SE number of genus-level taxa**	**Mean±SE number of family-level taxa**	**Phylum**	**Family**	**Genus**	**Shannon**	**Simpson**
100:0	9541	194 ± 4.17	303 ± 7.18	99.7	84.3	61.4	5.49	0.99
80:20	12267	182 ± 2.26	288 ± 4.38	99.9	84.5	60.3	5.47	0.99
60:40	10904	184 ± 4.08	290 ± 4.49	99.9	83.1	58.2	5.49	0.99
40:60	12267	181 ± 4.90	291 ± 6.04	99.9	82.0	57.2	5.46	0.99
20:80	12267	178 ± 4.41	283 ± 7.91	99.9	80.4	55.6	5.43	0.99
0:100	9541	182 ± 3.67	285 ± 6.32	99.9	83.3	56.4	5.47	0.99

### Gut community compositions

Bacteroidetes, Firmicutes and Proteobacteria dominated gut communities, but Synergistetes, Actinobacteria and Planctomycetes were also abundant, with the former particularly so for cockroaches fed on low amounts of fungus. Previous studies have established the predominance of Bacteroidetes and Firmicutes in cockroach guts and they commonly represent lineages shared amongst omnivorous animals [21, 26, 42]. Cockroaches on our 0% fungal diet were comparable to those of previous analyses on *P*. *surinamensis*, with a high abundance of Bacteroidetes, including families such as the Porphyromonadaceae, and Firmicutes such as the Lachnospiraceae [39] (S5 Table). Across the diet regimes, the 20 most abundant bacteria accounted for 35.2% of the total community abundance (S2 and S3 Tables), and these were five genus-level Firmicutes OTUs (12.1%), six genus-level Bacteriodetes OTUs (5.6%), five genus-level Proteobacteria OTUs (7.2%), two genus-level Actinobacteria OTUs (2.2%) and one OTU from each of the phyla Synergistetes (*Candidatus* Tammella; 3.6%) and Planctomycetes (Termite cockroach cluster 1; 3.3%) (S2 and S5 Tables).

### Gut community composition changes associated with a shift to a fungal diet

**Fig 4:**
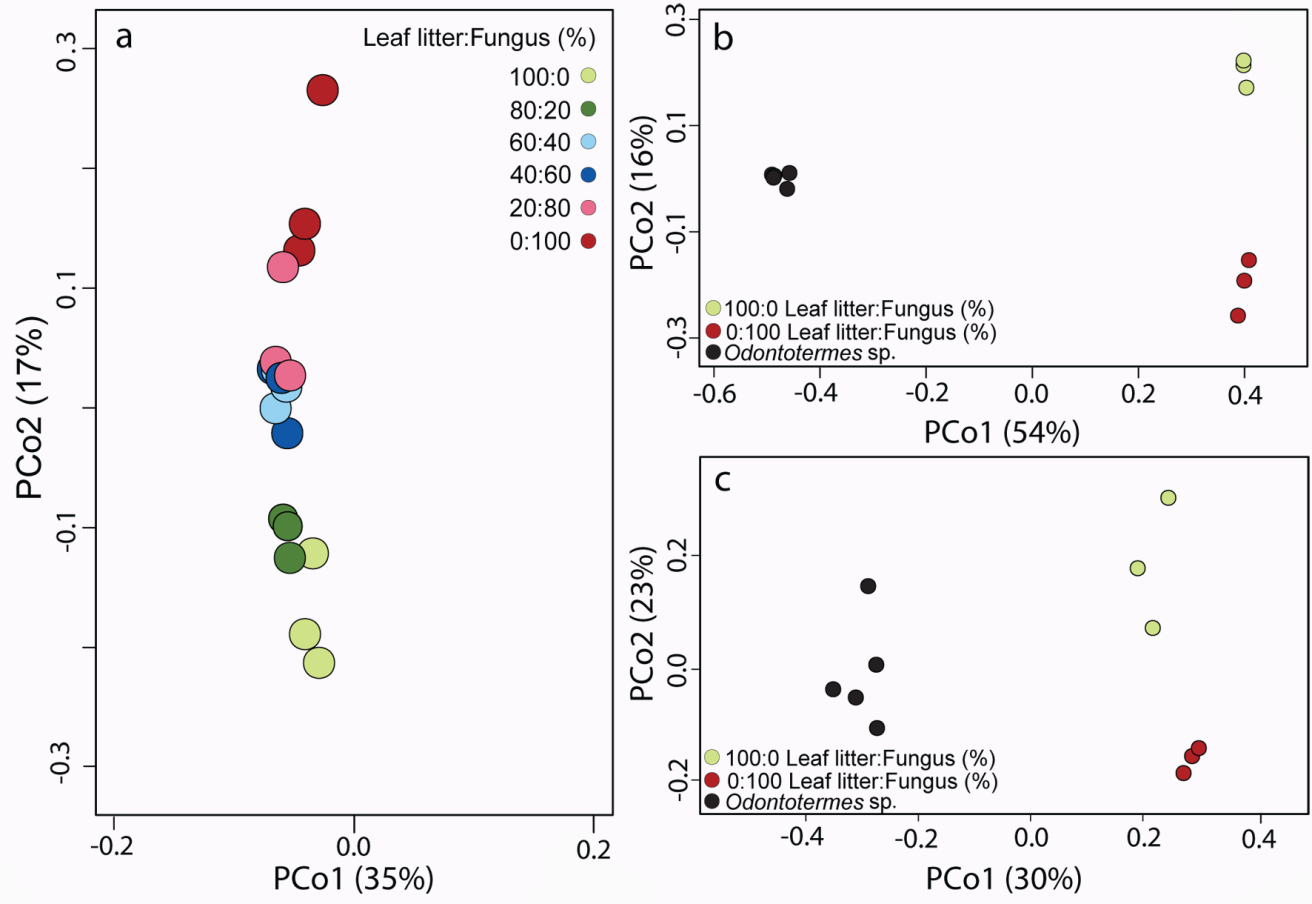
(a) PCoA similarity analysis of the three biological (averages of three technical replicates) replicates for each of the six fungal diets visualized via Bray-Curtis distances. S4 Table gives the PCoA loading values, and S5 Table lists the 20 bacteria that contribute the most to the separation between communities (b) PCoA similarity analysis visualized via Bray-Curtis distances across gut samples from cockroaches fed only 0% and 100% fungal diets, and including five samples from *Odontotermes* sp. that maintain the fungal species that was used in the feeding experiment. S6 Table gives the PCoA loading values, and S7 Table lists the 20 bacteria that contribute the most to the separation between communities. (c) PCoA similarity analysis visualized via Bray-Curtis distances including only bacterial OTUs present in both 0 and 100% fungal diets and *Odontotermes* sp. S8 Table gives the PCoA loading values, and S2 and S9 Tables list the 20 bacteria that contribute the most to the separation between communities.

Our comparison of bacterial community diversity in the guts of *P*. *surinamensis* cockroaches fed on increasing dietary proportions of fungal material demonstrated the influence diet can have as a structuring force of communities. We observed a remarkably clear signal of diet-specific effects on community structure, with microbiota from cockroaches fed on the same diet more similar to each other than to those from cockroaches on different diets (Fig 4A). This distinct step-wise gradient in community similarity from 0% to 100% fungus further implies that not only the presence but also the proportion of fungus in the diet shapes communities (Fig 4A). Using loading values from the PCoA analysis (S4 Table), we identified the OTUs that contributed the most to the pattern observed in Fig 4A and a heatmap of their abundances is given in S5 Table. Eighteen of these OTUs were also recovered in a similar analysis on which bacteria contribute to the separation of gut communities in cockroaches on 0% or 100% fungal diet (S8 and S9 Tables) and these OTUs are given in Table 2.

**Table 2:** The 20 genus-level taxa that contribute the most to the separation of 0% and 100% fungal diets, based on loading values for a PCoA analysis (S8 Table), and a comparison to the abundances of these bacteria in the gut microbiota of five colonies of *Odontotermes* sp. [36] (S9 Table).

**Genus-level classification**	**Average abundance in 0% fungal diet**	**Average abundance in 100% fungal diet**	**Average change in abundance in 100% fungal diet**	**Average abundance in *Odontotermes* sp.**
Planctomycetaceae, Termite cockroach cluster 1	7.0%	0.2%	-6.8%	Absent
*Candidatus* Tammella	3.8%	2.6%	-1.2%	0.01%
Unclassified Proteobacteria, Insect cluster II	3.3%	2.3%	-1.1%	Absent
*Sulfurospirillum*	0.8%	0.4%	-0.5%	Absent
Porphyromonadaceae Gut group, Termite cluster I	1.9%	1.6%	-0.3%	0.004%
*Actinomyces* 2	1.3%	1.3%	0.0%	Absent
Unclassified Lactobacillales	2.5%	2.5%	0.1%	0.0005%
Desulfovibrionaceae, Gut cluster 3	1.1%	1.5%	0.4%	Absent
Unclassified Lachnospiraceae	1.6%	2.0%	0.4%	0.009%
Porphyromonadaceae 3 Cluster IV	1.0%	1.6%	0.6%	Absent
Desulfovibrionaceae Gut cluster 3	1.6%	2.2%	0.6%	0.008%
Unclassified Betaproteobacteria	1.1%	1.7%	0.6%	0.004%
Ruminococcaceae, Termite cockroach cluster	0.6%	1.2%	0.6%	0.003%
*Tannerella*	0.7%	1.5%	0.9%	0.02%
Unclassified Porphyromonadaceae 3	0.3%	1.3%	1.0%	0.004%
Unclassified Micrococcales 3	0.03%	1.1%	1.0%	0.0002%
Unclassified Porphyromonadaceae 2	0.3%	1.6%	1.3%	0.02%
Unclassified Peptostreptococcaceae	0.02%	1.5%	1.5%	Absent
*Lactobacillus* 4	3.7%	5.2%	1.6%	Absent
*Weissella* 1	1.0%	2.7%	1.7%	Absent

**Fig 5:**
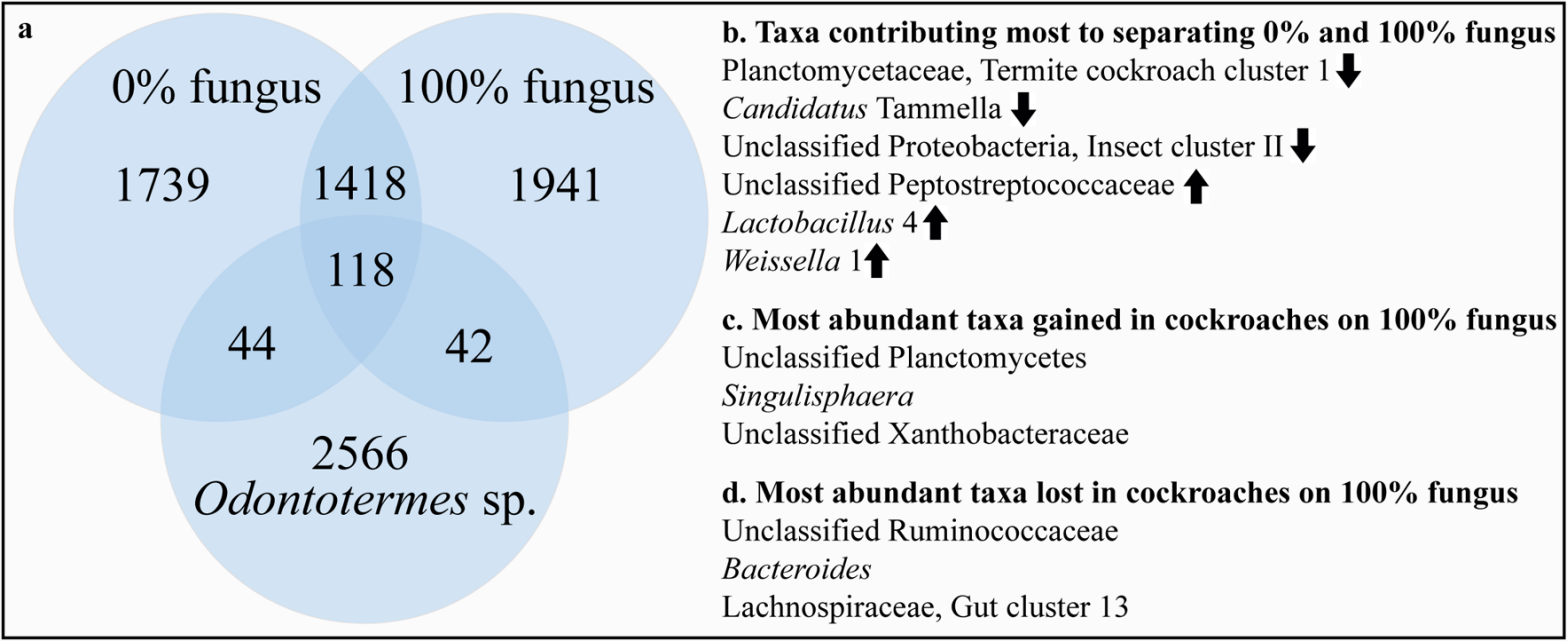
Summary of gut community changes associated with a shift from a leaf-litter to a fungal diet in *Pycnoscelus surinamensis*. (a) Venn diagram showing the shared and unique number of genus-level taxa identified in a combined analysis of gut microbial communities in cockroaches feeding on leaf litter (0% fungus), 100% fungus, and five colonies of *Odontotermes* sp. (b) Taxa contributing most to separating 0% and 100% fungus (full results in Table 2) (c) Most abundant taxa gained in cockroaches on 100% fungus (full results in Table 3) (d) Most abundant taxa lost in cockroaches on 100% fungus (full results in Table 4).

Five of the bacteria that contribute the most to this shift were reduced in average relative abundance in the 100% fungus diet, with the Termite cockroach cluster 1 (Planctomycetes) OTU exhibiting the most marked change from an average of 7.0% relative abundance in cockroaches on leaf litter to only 0.2% in the 100% fungal diet (Fig 5). The functional role of Planctomycetes in termite and cockroach gut environments is not well resolved [43], but it has been proposed that they may be involved in the breakdown of microbial polymers in decaying wood and humus [44, 45] which could conceivably be less abundant in a strictly fungal diet. The few other reduced taxa were *Candidatus* Tammella (1.2% average reduction), an unclassified Insect Cluster II OTU in the Proteobacteria; (-1.0%), *Sulfurospirillum* (-0.5%) and the OTU Termite cluster I in the Porphyromonadaceae Gut group (-0.5%). *Candidatus* Tammella has been identified primarily in lower termites as an obligate motility symbiont of gut flagellates in species such as the dry wood termite *Cryptotermes cavifrons* [46], as well as in the guts of cockroach families [21]. Relatives of this genus have also been isolated as a free-living bacterium and the genus itself has been identified as part of the core microbiota of higher termites, increasing in relative abundance within the fungus growing termites, suggesting an important function for these bacteria in the termite gut [22, 47, 48]. Although their function in higher termites remains unclear, there have been suggestions that bacteria within the *Synergistetes* are involved in amino acid fermentation in the termite gut, given the amino acid breakdown capabilities of this phylum and amino acid availability in the gut environment [46]. The observed decline of this genus in cockroaches fed on a 100% fungal diet is unexpected, given the previously recorded increase in its abundance in the fungus-growing termites [21, 22]. The reduced abundance of the genus within this study however may signal a decline in cockroach-specific lineages within the *Candidatus* Tammella genus, as they are forced onto a more fungal-based diet [21].

**Table 3:** The relative abundance of genus-level taxa present in some cockroaches feeding on 100% fungal diet and in *Odontotermes* sp., but not in cockroaches fed on a 0% fungal diet.

**Genus-level classification**	**Average in five colonies of *Odontotermes* sp.**	**Average in cockroaches on a 100% fungal diet**
Unclassified Planctomycetes	0.2868%	0.0240%
*Singulisphaera*	0.0013%	0.0233%
Unclassified Xanthobacteraceae	0.0004%	0.0219%
*Devosia*-*Prosthecomicrobium*	0.0067%	0.0145%
Unclassified Ruminococcaceae	0.0022%	0.0142%
Unclassified Lachnospiraceae	0.1450%	0.0141%
*Singulisphaera*	0.0023%	0.0105%
Ruminococcaceae, Insect cluster	0.0047%	0.0062%
Unclassified Verrucomicrobia	0.0052%	0.0057%
Ruminococcaceae, Termite cockroach cluster	0.0033%	0.0052%
*Marmoricola*	0.0039%	0.0046%
Unclassified Planctomycetes	0.0008%	0.0045%
Ruminococcaceae, Insect cluster	0.0025%	0.0042%
Unclassified Proteobacteria	0.0011%	0.0042%
*Dysgonomonas*	0.0726%	0.0041%
Ruminococcaceae, Termite cockroach cluster	0.0013%	0.0039%
Unclassified Planctomycetes	0.0008%	0.0038%
Ruminococcaceae, Termite cockroach cluster	0.0058%	0.0025%
Unclassified Firmicutes	0.0021%	0.0023%
Veillonellaceae, Uncultured 7	0.0022%	0.0022%
*Candidatus* Chloroacidobacterium	0.0008%	0.0022%
*Streptomyces* 1	0.0006%	0.0022%
Planctomycetaceae, Gut cluster 2	0.2323%	0.0020%
*Marmoricola*	0.0029%	0.0018%
*Pelomonas*	0.0010%	0.0018%
*Sphingomonas* 2	0.0051%	0.0015%
*Nocardioides*	0.0019%	0.0015%
Acidobacteriaceae, Uncultured 31	0.0017%	0.0015%
*Thermomonas* 2	0.0089%	0.0015%
Unclassified Firmicutes	0.0029%	0.0015%
*Patulibacter*	0.0026%	0.0015%
Unclassified Planctomycetes	0.0017%	0.0015%
Unclassified Firmicutes	0.0169%	0.0010%
Unclassified Actinobacteria	0.0017%	0.0010%
*Actinomadura* 1	0.0013%	0.0010%
Veillonellaceae, Uncultured 7	0.0011%	0.0010%
Unclassified Actinobacteria	0.0011%	0.0010%
*Solirubrobacter*	0.0010%	0.0010%
*Haliangium*	0.0006%	0.0010%
Ruminococcaceae. Insect cluster	0.0006%	0.0008%
Unclassified Ruminococcaceae	0.0050%	0.0008%
Xanthobacteraceae, Uncultured 1	0.0011%	0.0008%

**Table 4:** The identity and number of OTUs within genus-level taxa that were present in some cockroaches feeding on leaf litter (0% fungus), but absent in all cockroaches feeding on 100% fungal diet and their abundances across five colonies of *Odontotermes* sp. and cockroaches feeding on 0% fungus (only the 40 taxa that were most abundant in cockroaches feeding on 0% fungus are given, for the full results see S10 and S11 Tables).

**Genus-level classification**	**Number of OTUs**	**Sum of average abundances of OTU in *Odontotermes* sp.**	**Sum of average abundances of OTU in 0% fungus diet**
Unclassified Ruminococcaceae	164	0.0621	0.6805%
*Bacteroides*	11	Absent	0.5592%
Lachnospiraceae, Gut cluster 13	125	Absent	0.4197%
Unclassified Lachnospiraceae	83	Absent	0.3700%
Unclassified Clostridiales	92	0.0017	0.2682%
Ruminococcaceae, Termite cockroach cluster	70	0.0011	0.2594%
Unclassified Firmicutes	67	0.0046	0.2503%
Desulfovibrionaceae, Gut cluster 3	63	0.0013	0.2098%
Unclassified Porphyromonadaceae 3	69	Absent	0.1762%
Planctomycetaceae, Termite cockroach cluster 1	17	Absent	0.1720%
Ruminococcaceae, Insect cluster	32	0.0059	0.1671%
Planctomycetaceae, Termite cockroach cluster 2	34	Absent	0.1376%
Unclassified Bacteriodetes	41	Absent	0.1291%
Unclassified Lactobacillales	42	0.0027	0.1151%
Unclassified Porphyromonadaceae, Cluster V	41	Absent	0.1018%
*Alistipes* IV	32	0.0153%	0.0917%
Porphyromonadaceae 3, Cluster IV	24	Absent	0.0765%
*Dysgonomonas*	14	Absent	0.0706%
Unclassified Planctomycetaceae	25	Absent	0.0681%
Unclassified Porphyromonadaceae 2	2	Absent	0.0662%
Lachnospiraceae, Termite cluster	4	Absent	0.0625%
Porphyromonadaceae Cluster V, Termite Cockroach cluster	19	Absent	0.0623%
Unclassified Rikenellaceae	21	Absent	0.0561%
Porphyromonadaceae Cluster V, Cockroach cluster	10	Absent	0.0528%
*Catabacter*	8	Absent	0.0502%
*Tannerella*	21	Absent	0.0495%
Ruminococcaceae, Gut cluster 1	3	Absent	0.0491%
Ruminococcaceae, Gut cluster 4	3	Absent	0.0487%
Unclassified Proteobacteria	12	0.0045%	0.0470%
*Anaerotruncus*	8	0.0220%	0.0440%
Unclassified Nocardioidaceae	8	Absent	0.0435%
*Opitutus*	6	Absent	0.0433%
Ruminococcaceae, Gut cluster 9	2	0.0025%	0.0432%
*Candidatus* Tammella	11	Absent	0.0430%
Unclassified Mollicutes RF9	11	Absent	0.0426%
*Mucispirillum*	5	Absent	0.0412%
Lachnospiraceae, Gut cluster 15	4	Absent	0.0410%
Unclassified Corynebacteriales	9	Absent	0.0385%
*Candidatus* Arthromitus	10	Absent	0.0382%
Unclassified Clostridiales, Family XIII Incertae Sedis	12	Absent	0.0381%

Of the 14 OTUs that increased in abundance in cockroaches fed a 100% fungal diet, the Firmicutes *Weissella* 1 (1.7% increase), *Lactobacillus* 4 (1.6%) and an unclassified OTU in the Peptostreptococcaceae (1.5%) increased the most. Nine of these 14 OTUs were present in the samples from *Odontotermes* sp., but they were consistently low in relative abundances, with the most abundant taxa being *Tannerella* and an unclassified Porphyromonadaceae 2 (both present in only 0.02% relative abundance). However, several bacterial OTUs that were in low abundance in the original cockroach gut community may be closely related to *Odontotermes* sp. symbionts. For example, Desulfovibrio 3 (3.3% average abundance across nine termite species) and Ruminococcaceae gut cluster 1 (4.3%) help drive the pattern of community similarity between the fungus-growing termite core and cockroach gut communities [22]. Other bacteria that also increased in abundance in cockroaches fed on 100% fungus included the genus-level taxon *Clostridium* XI and the family Porphyromonadaceae. These bacteria are also found in lower abundances within the fungus-growing termite core [22] suggesting that these rare lineages found within *P*. *surinamensis* are promoted by a fungal diet and contribute to the overall patterns of community similarity.

### Taxa selected for or against in fungal-fed cockroaches

In addition to the OTUs that contribute the most to the shifts associated with a fungal diet, we identified forty-two bacteria that were absent in leaf-litter feeding cockroaches, but present in 100% fungus-feeding cockroaches and *Odontotermes* sp. (Table 3; Fig 5). Since the soil and fungal diet was sterile at the onset of our experiment, these resurging bacteria were most likely present in low abundance in the original gut microbiota of *P*. *surinamensis* and selected for on the strict fungal diet. Lineages that were promoted by fungal biomass included members of the *Desulfovibrio*, Ruminococcaceae, and Porphyromonadaceae. Members of these taxa are also found in the fungus-growing termite core microbiota [22], suggesting that they were selected for due to their capacity to break down fungal material. Again, these changes in the abundance of less dominant bacteria within the cockroach gut community contribute to the separation observed between the two dietary extremes in Fig 4B and 4C.

We also explored which bacteria were potentially lost as a consequence of a strict fungal diet. We extracted the OTUs that were absent in 100% diets and explored their abundances in cockroaches feeding on leaf litter and in *Odontotermes* sp. A remarkable 1,776 OTUs belonging to 286 genus-level classifications were absent from cockroaches feeding on 100% fungus (S10 Table). These OTUs collectively amounted to 7.1% of the total average abundance across the cockroaches feeding on 0% fungus, suggesting a substantial level of change. 94.6% of these OTUs were assigned to members of the phyla Firmicutes (863 OTUs), Bacteroidetes (354), Proteobacteria (207), Actinobacteria (137) and the Planctomycetes (120) (S10 Table). Only 44 of these OTUs (30 of the genus-level classifications) were present in the five colonies of *Odontotermes* sp., and they were consistently present in very low abundances (average 0.01%) (S10 Table). The 40 most abundant genus-level classifications in cockroaches on the 0% fungal diet and *Odontotermes* sp. are listed in Table 4 (for the full results, see S11 Table). These taxa collectively binned 1,235 of the putatively lost OTUs (69.5%) and accounted for 75.3% of the 7.1% relative abundance in cockroaches feeding on leaf litter (Table 4, S11 Table). Of these 40, only 11 were identified in *Odontotermes* sp., corroborating that the taxa reduced in fungus-feeding cockroaches are largely absent in the fungus-farming termites. It is unclear whether these bacterial lineages were lost permanently or whether they were merely reduced below the detection limit of our taxon classification approach and would re-establish if a lignocellulose-based diet was reintroduced.

Bacterial taxa that are reduced in fungus-fed cockroaches, such as members of the Clostridiales (Table 4), may decline as they lose functional importance due to the altered diet regime. Previous studies have revealed that genus-level lineages associated with the families of Lachnospiraceae, Porphyromonadaceae and Ruminococcaceae dominate the normal gut microbiota of omnivorous cockroaches [17, 28, 42]. While the OTUs present in cockroaches on a 0% fungus diet appeared absent or low in abundance in 100% fungus-fed cockroaches and in *Odontotermes* sp. (Table 4), it should be noted that other OTUs in these families are represented in *Odontotermes* sp. [22, 36]. These families are common in cockroaches on an omnivorous diet and in the guts of other insects, where they serve similar metabolic activities. Lachnospiraceae produce short chain fatty acids that provide the main carbon source for their insect host as lignocellulosic material is broken down [4]. A shift to a proteinaceous diet as cockroaches are fed on increasing proportions of fungal material may cause such bacteria to become functionally redundant and decline in abundance as alternative lineages able to utilize fungal biomass are promoted.

### A fungal diet alone does not make guts converge upon those of fungus-farming termites

The PCoA analysis comparing the two extremes, 0% and 100% fungal diet, with community similarities to *Odontotermes* sp. (Fig 4B) revealed that cockroaches fed on a 100% fungal diet did not approach *Odontotermes* sp. in similarity, as shown by their placement in PCoA space. To test whether this pattern was driven by bacteria absent in *P*. *surinamensis*, but present in relatively high abundance in *Odontotermes* sp. (e.g., *Alistipes* II (4.3% relative abundance), *Treponema* Ia (2.0%), and *Dysgonomonas* (1.8%); [38]), we conducted a PCoA including only bacterial taxa present in cockroaches on 0% and 100% fungal diets and in *Odontotermes* sp. and found that this was not the case (Fig 4C; S2 Table). In contrast, the main bacteria that constitute the *P*. *surinamensis* gut community, such as Enterococcaceae and Lactobacillaceae, remained in high abundances across all fungal dietary treatments, potentially because they remain important on a fungal diet or because they serve functions unrelated to digestion.

Our findings are consistent with previous work of resilience of microbial communities in the face of disruption in insect gut microbiotas [49, 50], including in cockroaches, in which a distinct core community is maintained even in the face of fundamental dietary shifts [27] (Fig 5). In addition to these evolutionary constraints, it is evident that factors other than diet shape community compositions in both cockroaches and farming termites. Germ-free studies in *S*. *lateralis* have shown the host gut environment plays a deterministic role in determining which bacterial lineages from the environment can colonize. Diet-related differences observed in the current study may also be explained by fundamental changes in the gut environment, which would be consistent with what has been suggested for higher termites [51]. We did not explore functional changes associated with the diet shift, but it is conceivable that enzymes involved in plant and fungal cell wall degradation would be vulnerable to such a shift, and that alterations to bacterial gene expression also may occur in bacteria with both suites of enzymes. Further experimentation using longer feeding periods, and providing cockroaches with fungus-growing termite bacterial inocula, could help establish the longer-term implications of exposing cockroaches to a fungal based diet and the extents to which diet can shape the gut microbiota.

### Conclusions

Our findings demonstrate, in a remarkably consistent step-wise manner, how a fungal diet can play a role in structuring gut community compositions in cockroaches, while exemplifying how original community compositions, and likely the inherent gut microenvironment, constrain the extent and magnitude of such change. Cockroaches assemble host-specific bacterial communities, just like termites do, but diet contributes to modulating the gut environment to provide a new set of colonisable (functional) niches and microhabitats for bacteria to colonize. This has been known for higher termites on longer evolutionary timescales, but our findings support that this is also apparent in cockroaches. The importance of the gut environment thus is a likely determinant of gut community structure to provide a mechanism connecting the digestive adaptations/diets to changes in community structure.

## Supporting information

S1 Table Gut samples selected for amplification and MiSeq sequencing. Target PCR products were visualized via agarose gel electrophoresis before submission to MiSeq. 1^st^ yield samples that were unable to be visualized clearly on a gel were run again using the 2^nd^ yield elution samples. Samples that still failed to display a significant banding pattern were diluted in order to counter any impurities present in the sample. DNA template samples were diluted to 1/10 and 1/50 of their original concentration with the additions of sterile distilled water and run using the same PCR conditions and visualised on an agarose gel. Samples that were then clearly visible on an agarose gel and therefore contained quantifiable DNA were submitted for MiSeq.
(XLSX)

S2 Table Relative abundance of taxa in the 16S rRNA libraries from *Pycnoscelus surinamensis* fed on different percentage ratio combinations of fungus (*Termitomyces*) and leaf litter. Classification results were obtained from sequence alignment against the manually curated reference database DictDb v.3 [39] and can be displayed for different taxonomic levels (Phylum; Class; Order; Family; Genus; Operational taxonomic units created at 98% sequence similarity).
(XLSX)

S3 Table Relative abundances of OTUs across the six diet treatments, averaged across three technical replicates. The average relative abundance of each OTU across the 18 biological replicates is shown.
(XLSX)

S4 Table Loading values of OTUs across the six diet treatments, averaged across technical replicates that contribute to the pattern observed in the PCoA in Fig 4A. Loading values were calculated via Principal Component analysis (PCA) of relative abundance data. The total contribution of each OTU to the pattern observed in the PCoA is calculated via the sum of loading values across all 18 principal components. OTUs are ordered according to their total contribution.
(XLSX)

S5 Table Heatmap of abundances of the 20 bacteria that based on loading values from the PCoA (S4 Table), contribute the most to the pattern observed in the PCoA in Fig 4A: the dataset including 18 biological replicates, averaged across technical replicates, for all six diet treatments. The heatmap scale is the percentage of reads assigned to a given taxon out of the total number of the high-quality filtered and classified reads for the treatment sample.
(XLSX)

S6 Table Loading values of OTUs that contribute to the pattern observed in the PCoA in Fig 4B, including gut samples from cockroaches fed only on 0% and 100% fungal biomass and including five samples from *Odontotermes* sp. Relative abundances from cockroach samples were averaged across technical replicates to give 3 biological replicates for each diet treatment. Loading values were calculated via Principal Component analysis (PCA) of relative abundance data. The total contribution of each OTU to the pattern observed in the PCoA is calculated via the sum of loading values across all 11 principal components. OTUs are ordered according to their total contribution.
(XLSX)

S7 Table Heatmap of abundances of the 20 bacteria that, based on loading values from the PCoA (S6 Table), contribute the most to the pattern observed in the PCoA in Fig 4B: the dataset including gut samples from cockroaches fed only on 0% and 100% fungal biomass and including five samples from *Odontotermes* sp. The heatmap scale is the percentage of reads assigned to a given taxon out of the total number of the high-quality filtered and classified reads for the treatment sample.
(XLSX)

S8 Table Loading values of OTUs that contribute to the pattern from a PCoA including gut samples from cockroaches fed on 0% and 100% fungal diets. Loading values were calculated via Principal Component analysis (PCA) of relative abundance data. The total contribution of each OTU to the pattern observed in the PCoA is calculated via the sum of loading values across all 7 principal components. OTUs are ordered according to their total contribution.
(XLSX)

S9 Table Abundances of the 20 bacteria that contribute the most to the observed shifts between 0% and 100% fungus diet and the associated mean abundance of these bacteria across the five *Odontotermes* sp. samples. (XLSX)

S10 Table Identity and relative abundance of OTUs that were present in cockroaches feeding on 0% fungus but absent in the 100% fungus diet treatment. (XLSX)

S11 Table Identity and relative abundance of OTUs that were present in cockroaches feeding on 0% fungus but absent in the 100% fungus diet treatment, summed over genus-level classification. (XLSX)
